# Measurement Method of Physical Parameters of Two-Phase Flow Based on Dual-Frequency Demodulation

**DOI:** 10.3390/s23239354

**Published:** 2023-11-23

**Authors:** Chunhui Song, Chengzhi Yao, Qinghong Liu, Wenyu Sun, Hui Zhang

**Affiliations:** Key Laboratory of Engineering Dielectrics and Its Applications, Ministry of Education, Harbin University of Science and Technology, Harbin 150080, China; songchunhui@hrbust.edu.cn (C.S.); yaochengzhi1026@foxmail.com (C.Y.); qhliu0601@163.com (Q.L.); 18752313952@163.com (W.S.)

**Keywords:** oil-water two-phase flow, physical property parameters, on-line measurement

## Abstract

Oil-water two-phase flow commonly occurs in the process of crude oil electric dehydration. Here, through dynamic changes in the water content and conductivity of oil-water two-phase flow in the process of electric dehydration, the influence of water content and conductivity on the efficiency and stability of electric dehydration is analyzed. Using real-time in-line measurements of water content and conductivity, the electric dehydration system is kept in an optimal state, which provides a basis for realizing efficient oil-water separation. Measurements of the physical parameters of oil-water two-phase flow is affected by many factors, such as the temperature of the two-phase flow, composition of the two-phase flow medium, structure of the measurement sensor, coupling of the conventional resistance–capacitance excitation signal, and processing of the measurement data. This complexity causes, some shortcomings to the control system, such as a large measurement error, limited measurement range, inability to measure the medium water phase as a conductive water phase, etc., and not meeting the requirements of the electric dehydration process. To solve that the conductivity and water content of high-conductivity crude oil emulsions cannot be measured synchronously, the RC relationship of oil-water emulsions is measured synchronously using dual-frequency digital demodulation technology, which verifies the feasibility of our test method for the synchronous measurement of physical parameters of homogeneous oil-water two-phase flow. Experimental results show that the novel measuring method (which is within the target measuring range) can be used to measure water content 0~40% and conductivity 1 ms/m~100 ms/m. The measuring error of the water content is less than 2%, and the measuring error of the conductivity is less than 5%.

## 1. Introduction

Oil-water two-phase flow is common in the process of oil and gas development and processing. It is very important to monitor its physical parameters in real time, especially in the process of electric dehydration, where the two parameters of conductivity and water cut (and the relationship between them) are key to establishing an effective dehydration electric field [1,2,3,4,5,6]. Therefore, this investigation studies the method and system of synchronous measurement of conductivity and water content by dual-frequency digital demodulation technology.

The on-line measurement technology of oil-water two-phase flow physical parameters can be divided into single-point measurements and tomography measurements. The single-point measurement is mainly used for single-parameter measurements, while the tomography measurement has developed multimodal fusion technology for multiparameter measurement [5,7]. However, the latter is complex in design, and the accuracy of the imaging system is lower than that of the former. This design still uses single-point measurement and, on this basis, draws lessons from the design idea of multimodal fusion.

For single-point measurements, the measurement system developed by Demori et al. has 40 cm protection sections at the head and tail ends of the sensor electrode part, which are used to eliminate the leakage current caused by the spurious between the conductive water phase and the outside world, realize the separation between the measurement part and the external pipeline, and eliminate the influence of the spurious effect [8]. By analyzing the equivalent complex impedance model of two-phase flow, Li and Soleimani proposed that high-frequency excitation will improve the dielectric characteristics of the dielectric and improve the measurement accuracy [9].

For multimodal fusion in tomography technology, Wang combined electrical capacitance tomography (ECT) with electrical resistance tomography (ERT) and designed a unified four-terminal rectangular measurement sensor to suppress the influence of parasitic impedance on measurement. The electrode is used to measure the full-range phase holdup of multiphase flow in oil production, and the measurement error is significantly reduced [10]. Under high-frequency excitation, Zhang combined ECT and MIT (magnetic inductance tomography) technology to measure the static imaging of multiphase medium distribution and reconstructed the image by using the conductivity information measured by MIT and the relative dielectric constant distribution obtained by ECT. Experiments show that this dual-mode imaging technique can measure gas/water distribution and oil/water distribution when water is used as the reference phase and the conductivity is lower than 0.5 S/m. When using ECT technology to measure a medium with a conductivity of 0.02 S/m, the ECT system reaches the measurement limit of spatial resolution, and, below this critical value, ECT can distinguish oil from air in water [11]. To realize the dual-mode unified sensor, Sun studied the measurement accuracy and nonlinear characteristics of ECT and ERT electrode sensors and designed a unified electrode sensor, which was excited in time by a gating switch, thus avoiding the interference caused by simultaneous excitation and achieving their goal of simplifying electrodes and achieving accurate measurement [12].

For the measurement of the physical parameters of oil-water two-phase flow in the process of electric dehydration, it is only necessary to measure the two-phase flow at the inlet and outlet at a single point [13,14]. At present, there is no multimodal fusion technology similar to the tomography method for single-point measurement. Therefore, this paper presents a method to measure the physical parameters (moisture content and conductivity) of two-phase flow based on dual-frequency demodulation. The feasibility of the measurement method is experimentally verified.

## 2. Dielectric Measurement Theory of Oil-Water Two-Phase Flow

Measurement accuracy when monitoring the physical parameters of oil-water two-phase flow is affected by many factors. To achieve accurate measurement, the influence of various factors on the physical parameters of two-phase flow is analyzed theoretically in this section.

### 2.1. Complex Dielectric Measurement Theory

When the plates of a flat capacitor are filled with an ideal lossless dielectric, and a sinusoidal voltage with frequency *ω* and amplitude *V* is applied to the capacitor, the current *I*_0_ flowing through the capacitor at this time is expressed as:

(1) $$I_{0}=j\omega C_{0}V$$

According to Equation (1), the applied voltage leads the current by 90°. If the plates of the capacitor are filled with dielectric with dielectric constant *ε*, the current *I* of the capacitor is expressed as:

(2) $$I=j\omega \varepsilon C_{0}V$$

**Figure 1:**
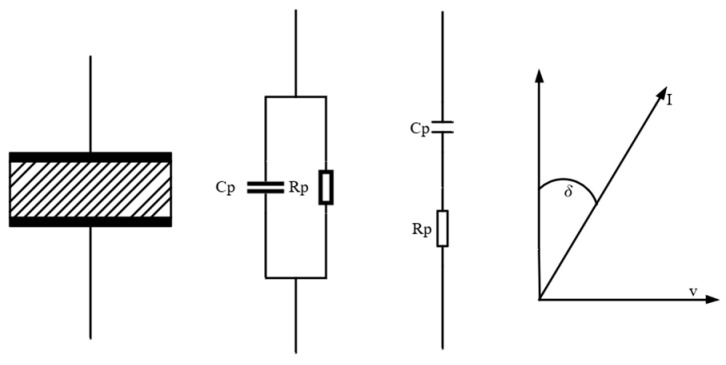
Capacitor filling medium and its equivalent model.

In a measurement process, the phase difference between the voltage and the current of the capacitor in the presence of medium is slightly less than 90° (as shown in Figure 1). We can decompose the current on the real axis and the imaginary axis, where the component on the real axis is expressed as $\omega \varepsilon^{\prime \prime }C_{0}V$, and the component on the imaginary axis is written as $\omega \varepsilon^{\prime }C_{0}V$. Then, the current flowing through the capacitor is expressed in another form:

(3) $$I=j\omega \varepsilon^{\prime }C_{0}V+j\omega \varepsilon^{\prime \prime }C_{0}V=j\omega \left(\varepsilon^{\prime }-j\varepsilon^{\prime \prime }\right)C_{0}V$$

Comparing Equation (2) with Equation (3) yields:

(4) $$\varepsilon =\varepsilon^{\prime }-j\varepsilon^{\prime \prime }$$

Equation (4) can be used to describe the complex dielectric constant, where the real part of the complex dielectric constant has the same meaning as the actual dielectric constant, and the real axis component of the current has the same direction as the voltage. It is expressed as a resistor connected in parallel with the capacitor to describe the dielectric loss.

### 2.2. Oil—Water Dielectric Properties

Pure oil is electron displacement polarized in an alternating electric field, and its setup time is 10^−15^~10^−16^ s, so the dielectric loss can be ignored, and its dielectric constant has nothing to do with the frequency of the applied electric field.

Water is a polar medium, and its polarization forms include electron displacement polarization and polar molecule turning polarization. The relationship between the dielectric constant and frequency of water is as follows:

(5) $${\varepsilon_{W}}^{\prime }=\frac{\varepsilon_{Ws}-\varepsilon_{W\infty }}{1+{\left(\omega \tau_{W}\right)}^{2}}+\varepsilon_{W\infty }$$

(6) $${\varepsilon_{W}}^{\prime \prime }=\frac{\left(\varepsilon_{Ws}-\varepsilon_{W\infty }\right)\omega \tau_{W}}{1+{\left(\omega \tau_{W}\right)}^{2}}+\frac{\sigma }{\omega \varepsilon_{Ws}}$$

In the formula, $\varepsilon_{Ws}$ is the static permittivity, $\varepsilon_{W\infty }$ is the infinite frequency permittivity, and $\tau_{W}$ is the water polarization time constant.

According to the experimental verification of a large number of scholars at home and abroad, the dielectric constant of oil is almost unaffected by temperature, which is generally between 2.0 and 3.0 [15,16,17]. Therefore, the temperature change of the dielectric constant of oil is not considered in this design.

The dielectric constant of water decreases with increasing temperature, and there the following relationship exists:

(7) $$\varepsilon =Ae^{-bt}$$

where *A* and *b* are constants. On the basis of summarizing the previous work, Ellison verified that the formula has high accuracy when *A* = 87.85306 and *b* = 0.00456992, and the error is less than 0.03 in the range of 0 °C~100 °C.

The equivalent dielectric model of two-phase flow is an exponential model as follows:

(8) $$\ln\varepsilon_{m}=\left(1-\alpha \right)\ln\varepsilon_{o}+\alpha \ln\varepsilon_{w}$$

where *ε*_m_ is the equivalent dielectric constant, *ε*_o_ is the dielectric constant of oil phase, and *ε*_w_ is the dielectric constant of water phase.

### 2.3. Equivalent Model of Two-Phase Flow

**Figure 2:**
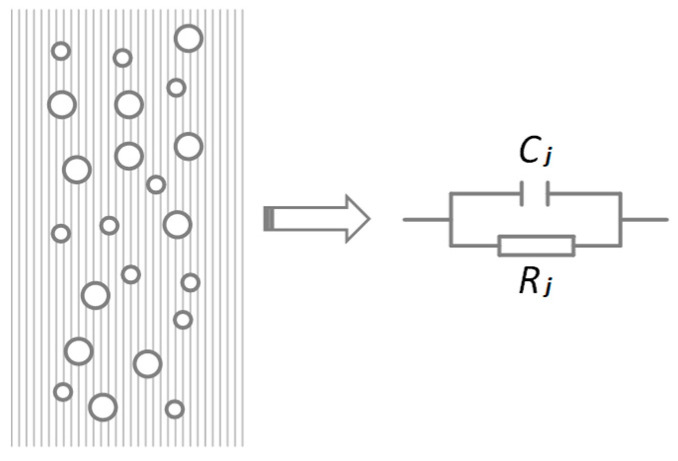
Equivalent model of the oil-water two-phase flow circuit.

Multiphase flow has both dielectric constant distribution characteristics and conductivity distribution characteristics, such that it is equivalent to the resistance–capacitance equivalent model in the measurement model (Figure 2).

According to the equivalent formula of two-phase flow parameters, the water content parameters in two-phase flow can be obtained by measuring the equivalent capacitance in the model, that is, measuring the displacement current. For the equivalent resistance model, combining it with the dielectric complex dielectric characteristic model, it can be known that the resistance model is the dielectric loss produced in the conductive water phase.

The loss is split into the relaxation loss caused by the steering polarization and the conductance loss caused by the dielectric conductance.

According to dielectric theory, the dielectric relaxation loss caused by relaxation polarization can be considered negligible in the total dielectric loss at high frequency.

Combined with the exponential model, the relationship between the electric field *E* and current density *J* is simplified as:

(9) $$J=\omega \varepsilon_{0}{\varepsilon_{W}}^{\prime \prime }E+j\omega \varepsilon_{0}{\varepsilon_{m}}^{\prime }E\approx \sigma E+j\omega \varepsilon_{0}{\varepsilon_{m}}^{\prime }E$$

The sensor output current is expressed as:

(10) $$I=\int_{\Omega }^{}J\cdot ds=\int_{\Omega }^{}\left(\sigma +j\omega \varepsilon_{0}\varepsilon_{m}\right)E\cdot ds$$

where Ω represents the effective conduction area of the sensor.

Current is the sum of the displacement current produced by the equivalent capacitance of two-phase flow and the conductivity current produced in the water phase. By measuring it, the equivalent dielectric constant and conductivity of the medium is obtained.

## 3. Measurement System Development

### 3.1. Analysis of the Influencing Factors of the Physical Parameters of Two-Phase Flow

To solve the problem that the conventional measuring device based on the capacitance measurement principle cannot measure the conductive water phase or the high phase holdup of the water phase, the traditional solution is to add insulating layers on the inner wall and outer wall of the sensor to suppress the conductive current and make the phenomenon of covering the displacement current disappear. However, the initial capacitance of the sensor is reduced, and the measurement difficulty is increased. In addition, to reduce the influence of the insulating layer on the measurement accuracy, higher technological requirements are put forward for the thickness and uniformity of the insulating layer. At the same time, the thickness of the insulating layer becomes thinner, which will lead to a decline in its corrosion resistance, which makes the accuracy of the sensor gradually decline with time. Therefore, to solve this problem, Wang used insulating ceramics with a relatively large relative dielectric constant as the insulating layer, but this method has an obvious cost increase, low adhesion, and poor practicability [18].

To realize the synchronous measurement of moisture content and conductivity, this paper presents a measurement method based on the principle of complex dielectric measurement. If the appropriate measurement circuit is matched to realize the independent measurement of the conduction current and displacement current, then the problems caused by the insulation layer can be avoided.

In this design, the principle of capacitance measurement is used for reference, and the dual-mode fusion technology of the electrical tomography system is used for reference. To meet the needs of synchronous measurements and unified sensors, a resistance-capacitance measurement circuit based on the impedance measurement principle is selected. A resistance–capacitance measurement circuit based on the impedance measurement principle, which is realized by in-phase demodulation and quadrature demodulation. The conduction current and displacement current are detected independently, which ensures the reliability and accuracy of the measurement.

**Figure 3:**
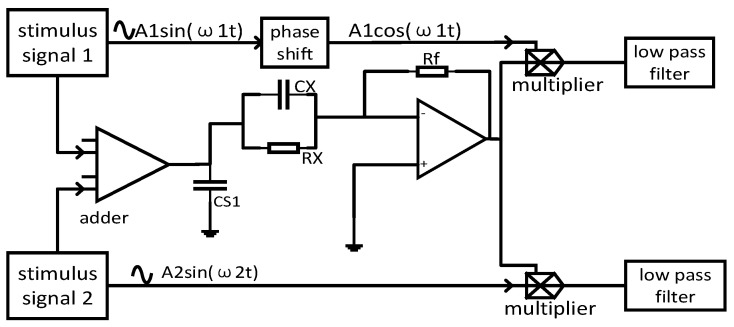
Resistance–capacitance measurement circuit based on impedance measurements.

**Figure 4:**
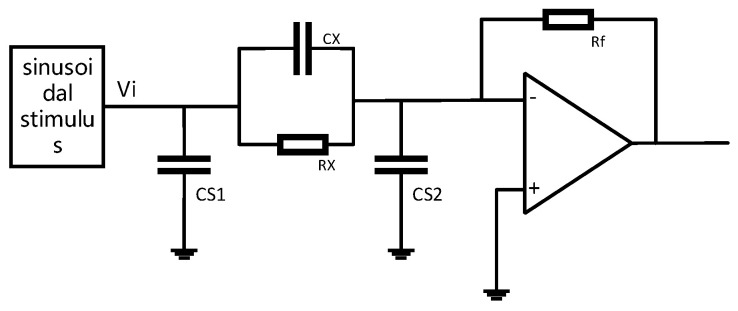
Basic impedance measurement circuit.

The specific circuit is shown in Figure 3 and Figure 4.

**Figure 5:**
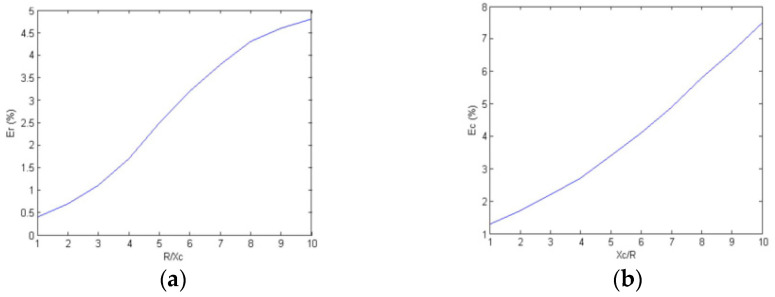
Resistance–capacitance mismatch measurement error. (**a**) Relative measurement error of resistance; (**b**) Relative measurement error of capacitance.

The above circuits are fabricated, and the corresponding measurement results and errors are analyzed. In this testing, the excitation frequency is uniformly fixed at 2 MHz. Measuring resistance and capacitance with different proportions, the measurement error is shown in Figure 5.

As shown in Figure 5, the measurement error is nearly proportional to the ratio of the resistance and capacitance reactance, and the error is minimized when the impedance and capacitance reactance are nearly equal. This occurs because when the ratio of resistance-capacitance-reactance increases, the greater the gap between the conduction current flowing through the resistor and the displacement current flowing through the capacitor, the greater the demodulation error.

When analog demodulation is used in the demodulation circuit, it is found that there is inherent DC bias at the output of the analog multiplier chip, which is approximately 20 mV. When the amplitude of the excitation signal is small, the error caused by DC bias cannot be ignored. In addition, the DC bias is related to the amplitude and frequency of the excitation signal, chip temperature drift and zero drift, so it cannot be regarded as a fixed value and directly eliminated from the output value.

### 3.2. Principle Derivation of a Two-Phase Flow Physical Parameter Measurement Circuit Based on the Impedance Measurement Principle

In view of the above-mentioned problems, the following schemes are proposed for improvement. For the error caused by resistance–capacitance error, automatic frequency conversion is used to realize resistance-capacitance matching. For the inherent defects of analog multiplier chips in analog phase-sensitive demodulation, a digital multiplication phase-sensitive demodulation scheme is adopted. Combined with the above schemes, a dual-frequency excitation digital multiplication demodulation method based on dielectric measurement theory is proposed. The theoretical derivation of this method is as follows:

Assume that the DAC unit outputs two channels and two frequencies *k*_1_*ω*. Superposition of *ω* and *k*_2_*ω* The excitation signals are as follows:

(11) $$V_{i}=A_{1}sin\left(k_{1}\omega t\right)+A_{2}\sin\left(k_{2}\omega t+\phi \right)$$

Combined with the measurement circuit shown in Figure 4, the output of the impedance detection circuit after AD conversion is as follows:

(12) $$\begin{array}{cc} V_{o}\left(n\right)= & -\frac{R_{f}}{R_{x}}[A_{1}\sin\left(\frac{2k_{1}\pi }{N}\right)+A_{2}\sin\left(\frac{2k_{2}\pi }{N}n+\varphi \right)]\\ & -R_{f}C_{X}[A_{1}\frac{2k_{1}\pi }{N}\sin\left(\frac{2k_{1}\pi }{N}n\right)+A_{2}\frac{2k_{2}\pi }{N}\sin\left(\frac{2k_{2}\pi }{N}n+\varphi \right)] \end{array}$$

where the signal amplitude at *A*_1_ and *A*_2_ is the phase angle of the signal, N corresponds to the value range of 0~*N* − 1, N is the number of sampling points, and *k*_1_ and *k*_2_ are assigned values to highlight different frequencies.

The reference signal of the two signal digital multipliers is expressed as:

(13) $$V_{fR}\left(n\right)=A_{2}sin(\frac{2k_{2}\pi }{N}n+\phi )$$

(14) $$V_{fC}\left(n\right)=A_{1}cos(\frac{2k_{1}\pi }{N}n)$$

Substituting Equation (12) into (13) and (14) yields:

(15) $$\begin{array}{cc} V_{OR}\left(n\right)= & -\frac{A_{2}R_{f}}{R_{X}}[(\frac{A_{1}}{2}cos\frac{2k_{1}\pi }{N}n-\frac{2k_{2}\pi }{N}n-\phi )-\frac{A_{1}}{2}\cos\left(\frac{2k_{1}\pi }{N}n+\frac{2k_{2}\pi }{N}n-\phi \right)\\ & +\frac{A_{2}}{2}-\frac{A_{2}}{2}cos(\frac{4k_{2}\pi }{N}n+2\phi )]-A_{2}R_{f}C_{X}[\frac{A_{1}k_{1}\pi }{N}\sin\left(\frac{2k_{1}\pi }{N}n+\frac{2k_{2}\pi }{N}n+\phi \right))\\ & +\frac{A_{1}k_{1}\pi }{N}\sin\left(\frac{2k_{1}\pi }{N}n-\frac{2k_{2}\pi }{N}n-\phi \right)+\frac{A_{2}k_{2}\pi }{N}\cos\left(\frac{4k_{2}\pi }{N}n+2\phi \right)] \end{array}$$

(16) $$\begin{array}{cc} V_{oC}\left(n\right)= & -\frac{A_{1}R_{f}}{R_{X}}[\frac{A_{1}}{2}\sin\left(\frac{4k_{1}\pi }{N}n\right)+\frac{A_{1}}{2}\cos\left(\frac{2k_{1}\pi }{N}n+\frac{2k_{2}\pi }{N}n+\phi \right)\\ & -\frac{A_{1}}{2}\sin\left(\frac{2k_{1}\pi }{N}n-\frac{2k_{2}\pi }{N}n-\phi \right)]-kA_{1}R_{f}C_{X}[\frac{A_{1}k_{1}\pi }{N}\cos\left(\frac{4k_{2}\pi }{N}n\right)+\frac{A_{1}k_{1}\pi }{N}\\ & +\frac{A_{2}k_{2}\pi }{N}\cos\left(\frac{2k_{1}\pi }{N}n+\frac{2k_{2}\pi }{N}n+\phi \right)+\frac{A_{2}k_{2}\pi }{N}\cos\left(\frac{2k_{1}\pi }{N}n-\frac{2k_{2}\pi }{N}n-\phi \right) \end{array}$$

The output of the digital multiplier is processed by low-pass filtering, and only the DC component containing resistance and capacitance information is retained:

(17) $$V_{oR}^{\prime }=\frac{A_{2}^{2}R_{f}}{2R_{x}}$$

(18) $$V_{oC}^{\prime }=\frac{A_{1}^{2}k_{1}\pi R_{f}C_{x}}{N}$$

The equivalent resistance and capacitance are:

(19) $$R_{x}=\frac{A_{2}^{2}R_{f}}{2V_{oR}^{\prime }}$$

(20) $$C_{x}=\frac{NV_{oC}^{\prime }}{A_{1}^{2}\pi k_{1}R_{f}}$$

Equations (19) and (20) indicate that the resistance is only related to the amplitude of the second output of the signal generating unit but not to the frequency and initial phase and that the capacitance is related to the amplitude and frequency of the first output of the signal generating unit but not to the amplitude and frequency of the second signal. Both can be acquired simultaneously without loss of information.

### 3.3. Hardware and Software Design of the Measurement System Circuit

**Figure 6:**
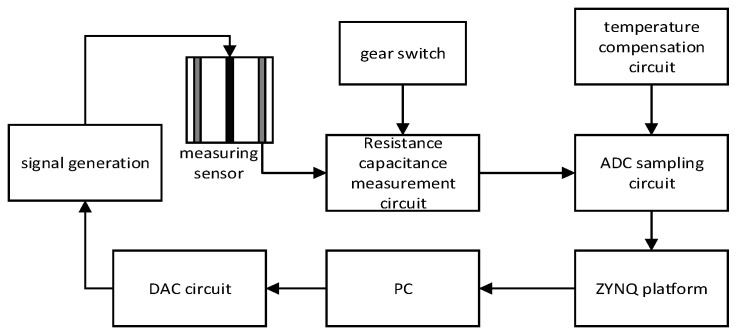
System design scheme of the measurement system.

The actual circuit of the above circuit is verified, and the system block diagram of the system measurement circuit is shown in Figure 6.

The components used in this measurement circuit are shown in Table 1.

In this design, ZYNQ is used as the measurement system MCU, which combines an FPGA with ARM, FPGA with DAC and ADC modules to complete excitation signal sending and resistance–capacitance signal acquisition, and demodulation of signals in FPGA. With this platform, automatic gear switching and resistance–capacitance matching are realized with ARM. The upper computer interface is used to display physical parameters in real time and manually adjust excitation signals. See Table 2.

Because the equivalent resistance and capacitance of the medium to be measured vary over multiple orders of magnitude under different water contents and salinities, it is necessary to design the feedback resistance in multiple positions. When using empirical formulas to idealize the feedback resistance, the optimal design parameters are adopted for the sensor structure parameters. The equivalent dielectric constant is selected as the exponential model, the equivalent conductance is selected as the Maxwell model, and the equivalent resistance and capacitance are calculated under the conditions of a water content of 0~40% and conductivity of 0 ms/m~100 ms/m. The equivalent capacitance ranges from 13.96 pF to 122.26 pF and corresponds to a 20 kΩ to 200 Ω resistor. Therefore, a six-gear switching circuit is designed. The feedback resistor fully considers the voltage input and output range of AD8033 and the input range of the ADC module and selects a six-gear feedback resistor.

## 4. Comparison and Verification of Measurement Results

### 4.1. Comparison of Resistance and Capacitance Measurement Results

The resistance and capacitance measurement results of the dual-frequency digital multiplication demodulation, traditional single-frequency analog phase-sensitive demodulation, dual-frequency analog phase-sensitive demodulation and single-frequency digital multiplication demodulation are verified. These measurement results uniformly adopt a 6-bit semidigital DM3068 as the measurement standard, and an error comparison of the results is shown in Table 3, Table 4, Table 5 and Table 6.

The above measurements are based on the following conditions:

(1) The amplitude of the excitation signal is 1 V.
(2) The frequency of the single-frequency excitation signal is 2 MHz.
(3) The dual-frequency excitation signal resistance excitation is 10 kHz, and the capacitance excitation frequency is automatically adjusted according to the principle of resistance–capacitance matching.
(4) The multiplier used for analog multiplication demodulation is AD734, which is not compensated for by other components or techniques.

The measurement error of digital demodulation is less than that of analog demodulation, and the measurement error of dual-frequency excitation is less than that of single-frequency excitation. This result is completely consistent with the theoretical derivation.

Digital demodulation technology can effectively solve the DC bias of a traditional analog demodulation chip, the multiplier’s nonlinearity, and the multiplier’s own accuracy, while digital demodulation depends more on the accuracy of AD conversion, which is a good solution at present.

### 4.2. Measurement and Verification of Physical Parameters of Oil-Water Two-Phase Flow

**Figure 7:**
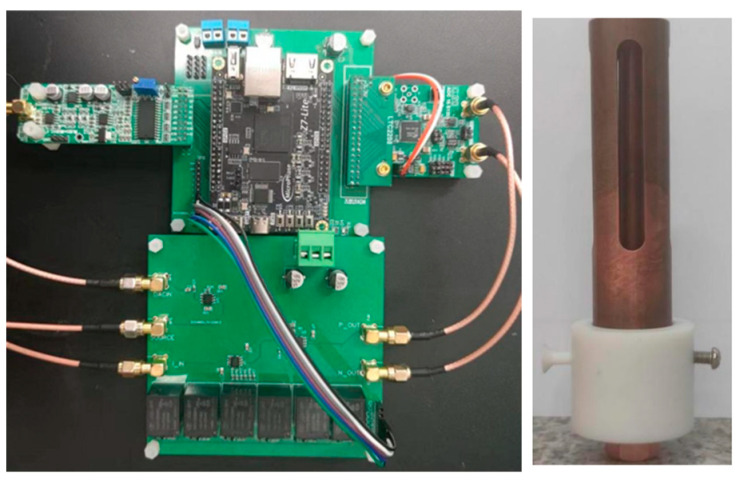
Physical diagram of the measurement system.

To verify the accuracy of the physical parameter measurements, two-phase flow solutions with different conductivities and water contents were proportioned, and the existing water content measuring instrument and crude oil conductivity measuring instrument were used as verification standards for water content and conductivity measurements. Brine with conductivities of 1.288 us/m, 11.13 ms/m, 14.13 ms/m, 14.6 ms/m, and 128.8 ms/m was used as the conductive water phase, 10# white oil was used as the oil phase, 21 groups of oil-water two-phase flows with different water contents and conductivities were mixed, and the emulsifier Span80 was added and stirred at 6000 rad/min for 5 min by a high-speed shearing machine to make it homogeneous and then measured. The physical diagram of the measurement system is shown in Figure 7. The measurement results are obtained by nonlinear fitting of data based on the BP neural network on the basis of resistance and capacitance measurements.

Table 7 and Table 8 show that the dual-frequency excitation digital multiplication demodulation method based on dielectric measurement theory combined with the BP neural network has a resistance–capacitance detection error of less than 2%, a moisture content measurement error of less than 1.1%, and a conductivity measurement error of less than 5%, which reflects that this method has a good resistance–capacitance measurement effect.

**Figure 8:**
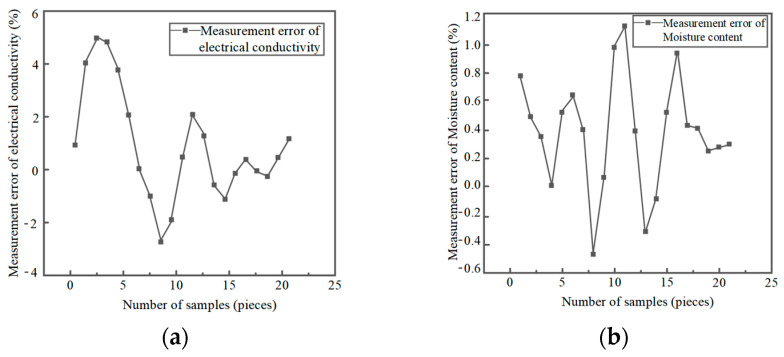
Dynamic measurement error of physical parameters. (**a**) Conductivity measurement error; (**b**) Moisture content measurement error.

The measurement error of the dynamic measurement of moisture content and conductivity is shown in Figure 8.

## 5. Conclusions

Based on the theory of dielectric measurement, a novel method for measuring physical parameters of oil-water two-phase flow with dual-frequency excitation and digital multiplication demodulation is presented. The main conclusions are summarized as follows:

1. Based on complex dielectric measurement theory and a resistance–capacitance equivalent model of two-phase flow, the feasibility of using complex dielectric measurement theory to measure the physical parameters of oil-water two-phase flow is verified.
2. Comparing the dual-frequency digital demodulation method with the traditional single-frequency analog demodulation method, the measurement error for resistance and capacitance is less than 2%, the measurement error for water cut is less than 1.1%, and the measurement error for conductivity is less than 5%, which verifies the broad prospect of this method in the online detection of physical parameters of oil-water two-phase flow.

## Figures and Tables

**Table 1:** Parameters of the simulation model.

Component Name	Model
MUC platform.	ZYNQ7100
DAC chip.	AD9764
ADC chip.	LTC2208
Operational amplifier of resistance–capacitance measuring circuit.	AD8033
Temperature sensor.	T10S-B-F
Measuring sensor	Coaxial cylindrical sensor with long 100 mm outer diameter 10 mm inner diameter 4 mm without insulating layer

**Table 2:** Gear design and scope of application.

File Serial Number	Feedback Resistance (Ω)	Applicable Resistance Range (Ω)	Applicable Capacitance Range (pf)
R1	500	200–1 k	220 pF–300 pF
R2	1.5 k	1 k–2 k	180 pF–220 pF
R3	2.2 k	2 k–3 k	140 pF–180 pF
R4	3.48 k	3 k–4.5 k	70 pF–140 pF
R5	5 k	4.5 k–8 k	40 pF–70 pF
R6	10 k	8 k–20 k	27.5 pF–40 pF

**Table 3:** Resistance-capacitance measurement results and errors.

Resistance and Capacitance to Be Measured	Measured Resistance Value (kΩ)	Capacitance Measured Value. (pF)	Resistance Error. (%)	Capacitance Error (%)
495.3 Ω/197.3 pF	0.536	182.7	8.2	−5.36
1.51 kΩ/49.6 pF	1.60	52.8	5.7	6.36
3.03 kΩ/22.3 pF	3.22	21.3	6.27	−4.28
4.98 kΩ/12.1 pF	5.15	12.7	3.48	4.68
10.1 kΩ/15.3 pF	10.6	16.1	4.74	5.35

**Table 4:** Measurement results and errors of dual-frequency analog demodulation resistance and capacitance.

Resistance and Capacitance to Be Measured	Measured Resistance Value (kΩ)	Capacitance Measured Value. (pF)	Resistance Error. (%)	Capacitance Error (%)
495.3 Ω/197.3 pF	0.516	190.7	4.2	−3.36
1.51 kΩ/49.6 pF	1.56	51.4	3.3	3.72
3.03 kΩ/22.3 pF	2.93	22.9	−3.2	2.84
4.98 kΩ/12.1 pF	5.12	12.5	2.72	3.32
10.1 kΩ/15.3 pF	9.88	14.9	−2.17	−2.78

**Table 5:** Measurement results and errors in the resistance and capacitance of single frequency digital demodulation.

Resistance and Capacitance to Be Measured	Measured Resistance Value (kΩ)	Capacitance Measured Value. (pF)	Resistance Error. (%)	Capacitance Error (%)
495.3 Ω/197.3 pF	0.526	187.0	6.2	−5.26
1.51 kΩ/49.6 pF	1.45	51.7	−3.86	4.27
3.03 kΩ/22.3 pF	2.90	27.3	−4.18	2.22
4.98 kΩ/12.1 pF	5.10	11.7	2.42	−3.02
10.1 kΩ/15.3 pF	10.4	15.9	3.17	4.22

**Table 6:** Resistance–capacitance measurement results and errors

Resistance and Capacitance to Be Measured	Measured Resistance Value (kΩ)	Capacitance Measured Value. (pF)	Resistance Error. (%)	Capacitance Error (%)
495.3 Ω/197.3 pF	0.496	195.4 pf	0.16	−0.96
1.51 kΩ/49.6 pF	1.54	49.2 pf	1.98	−0.806
3.03 kΩ/22.3 pF	3.07	22.6	1.32	1.34
4.98 kΩ/12.1 pF	4.99	12.3	0.2008	1.65
10.1 kΩ/15.3 pF	10.2	15.6	0.495	1.96

**Table 7:** Static measurement results and errors of moisture content and electrical conductivity.

Serial Number	Conductivity Measurement (mS/m)	Conductivity Calibration Value (mS/m)	Moisture Content Measurement (%)	Moisture Calibration Value (%)	Conductivity Error (%)	moisture Content Error (%)
1	0.98	0.971	9.4	9.329	0.912	0.76
2	1.12	1.07	10.5	10.45	4.11	0.48
3	1.26	1.20	11.6	11.56	4.99	0.34
4	1.4	1.33	12.6	12.6	4.93	0
5	1.54	1.48	13.7	13.63	3.83	0.51
6	1.68	1.64	14.3	14.21	2.08	0.63
7	1.82	1.82	15.4	15.34	0	0.39
8	1.96	1.98	16.5	16.58	−1.07	−0.48
9	2.1	2.16	17.5	17.49	−2.86	0.06
10	2.24	2.28	18.8	18.62	−1.96	0.96
11	2.38	2.37	19.9	19.68	0.46	1.11
12	2.52	2.47	20.8	20.72	2.14	0.38
13	2.66	2.63	21.6	21.669	1.28	−0.32
14	2.8	2.82	22.7	22.72	−0.64	−0.09
15	2.94	2.98	23.5	23.38	−1.19	0.51
16	3.08	3.09	25.1	24.87	−0.19	0.92
17	3.22	3.21	26.2	26.09	0.37	0.42
18	3.36	3.36	27.3	27.19	−0.09	0.4
19	3.5	3.51	28.6	28.53	−0.31	0.24
20	3.64	3.62	29.8	29.72	0.44	0.27
21	3.78	3.74	30.9	30.81	1.14	0.29

**Table 8:** Results and errors of the dynamic measurement of moisture content and electrical conductivity.

Physical Property Parameters to Be Measured	Measure Moisture Content (%)	Measure Conductivity (ms/m)	Moisture Content Error (%)	Conductivity Error (%)
40.1%/4 ms/m	40.15	4.1	0.12	2.5
20.6%/2.6 ms/m	20.68	2.7	0.38	3.8
10.3%/2 ms/m	10.41	2.08	1.05	4
9.5%/1.8 ms/m	9.59	1.76	0.94	2.22
4.7%/0.95 ms/m	4.72	0.91	0.42	4.2

## Data Availability

Data are contained within the article.
